# Exploring the Heart Failure Connection in Long COVID Patients: A Narrative Review

**DOI:** 10.7759/cureus.58694

**Published:** 2024-04-21

**Authors:** Emmanuel Olumuyide, Chibuike C Agwuegbo, Eman N Ahmed

**Affiliations:** 1 Internal Medicine, Advocate Illinois Masonic Medical Center, Chicago, USA; 2 Internal Medicine, Southwest Healthcare, Temecula, USA; 3 Internal Medicine, Alfaisal University College of Medicine, Riyadh, SAU

**Keywords:** wuhan coronavirus, sars-cov-2, 2019-ncov, cardiovascular/cardio and vascular/blood vessel disease, cardiac complications, covid-19, heart failure, long covid

## Abstract

In this narrative review, we explore the relationship between long COVID patients and their risk of developing heart failure (HF). Patients with long COVID face a heightened risk of HF, a critical cardiovascular complication linked to the prolonged effects of COVID-19. Clinical manifestations of long COVID-associated HF present diagnostic challenges, complicating patient management. Multidisciplinary care is essential to address these complexities effectively. We found that long COVID can result in various cardiovascular issues including HF. The current view is long COVID leads to HF by activating systemic inflammation by causing endothelial dysfunction, which leads to activation of the complement pathways, tissue factor pathways, and Von Willebrand factor; activation of all these factors leads to venous and arterial thrombosis, which could lead to clogging of blood vessel of the heart leading to cardiovascular complications. The association between long COVID and HF can be challenging despite being recognized as comorbidity because biomarkers are not dependable in determining whether a patient had HF before or after contracting COVID-19. Emerging therapeutic modalities offer hope for improving outcomes, but further research is needed to refine management strategies and mitigate long-term cardiovascular consequences of COVID-19.

## Introduction and background

The universal definition of heart failure (HF) was established in 2021, described as a condition resulting from an abnormality in the heart and indicated by elevated BNP levels and evidence of cardiogenic congestion. This definition, along with the categorization of HF into HFrEF (HF with reduced ejection fraction), mildly reduced, and HFpEF (HF with preserved ejection fraction), agrees with the 2021 ESC Guidelines on HF. Studies have shown that 2% of all patients hospitalized with COVID-19 developed HF, most commonly in patients with a history of other cardiovascular diseases, diabetes, obesity, and underlying peripheral artery disease. Incident acute HF was recognized as a complication in 2%, and myocardial injury in 10% of all patients hospitalized with COVID-19 [1].

Towards the end of 2019, we saw an emerging increase in pneumonia cases in Wuhan, China with unknown etiology. Weeks later, in January 2020, lower respiratory tract sample analyses via deep sequencing analysis led to the diagnosis of a novel virus severe acute respiratory syndrome coronavirus 2 (SARs-CoV-2) as the causative agent for the observed pneumonia cluster [2]. Long COVID refers to the persistence of various symptoms weeks to months after acquiring SARS-CoV-2 infection, irrespective of the viral status [3]. This condition is also called "post-COVID syndrome" and can manifest as continuous, or relapsing, and remitting symptoms. Individuals with this syndrome can experience the continuation of one or more symptoms of acute COVID-19 or an emergence of new symptoms. Scientists are yet to understand the exact pathophysiology leading to long COVID [1-3]. Long COVID has led to various deleterious effects in formerly healthy people with no past cardiovascular problems with new signs of HF. Unfortunately, the distinction between de-novo or preexisting clinical HF is impossible in most cases of long COVID [4]. The purpose of this paper is to provide an overview of the current understanding of the association of long COVID with HF.

## Review

Epidemiology 

Studies have also noticed 0.6% new diagnoses of HF in patients with COVID-19 of which 22% had no predisposing risk factors; however, the overall point prevalence for new-onset HF is low. These patients also showed a higher propensity for ICU admissions (32%) and intubation (24%) compared to non-HF cohorts [5]. Some of these patients would also develop cardiogenic shock [6]. Recent analytical evidence suggests a two times increased risk of de-novo cardiovascular problems with a 71% higher risk of HF associated with COVID-19 [7]. In an analysis from the US Department of Veterans Affairs (VA), they found that 2% of these patients had de-novo HF upon annual follow-up, with a need for readmission to revamp medications [8]. Nuzzi et al. worked on the same dataset and reported new onset right-sided failure was seen in 2.7% with no prior history of hypertension or left HF [8]. Others worked on the same dataset and noted HF to be one of the complications seen within 30 days to 12 months of acute COVID-19 infection. Specifically, HF and atrial fibrillation were seen in 10 extra patients within every 1000 patients, reflecting a huge burden [9]. Analysis of large-scale data from the N3C enclave was carried out to eliminate the demographic constraint of the VA dataset and they revealed 10979 cases of incident HF in patients infected approximately a year earlier [10]. Wang et al. utilized the TriNetX database and found the incidence of HF (HR=2.296) to be higher in COVID-19 survivors compared to non-infected controls [11]. In a meta-analysis assessing the risk of incident HF, they found an incidence of 1.1%. These survivors had a 90% risk of developing HF in the long-term period, which is directly proportional to the patient's age and history of hypertension [12]. Hospitalization for COVID-19 was the sole factor propelling higher incidence of incident HF (2.3% vs. 1.5% in control), mortality (3.3% vs. 2.6%), and composite death and incident HF (5.2% vs. 4.0%) observed post-discharge. These patients with incident HF were found to have shorter mean follow-up post-discharge (69 vs. 84 days) [10]. In a retrospective one-year follow-up study, out of the 54 patients with Major Adverse Cardiac Events (MACE), five were hospitalized due to HF [13].

Mechanisms

Unudurthi et al. describe various mechanisms that potentiate cardiac malfunction due to COVID-19. The viral content has the propensity to induce an inflammatory, hypercoagulable, lymphopenic, neutrophilic state and endothelial necrosis, hypoxia-mediated injury presentation that directly and indirectly affects the heart. This adds to the myocardial burden and can lead to HF [14]. The viral surface spike protein S uses its receptor binding domain to bind with the cellular receptor of ACE2 [15,16]. The transmembrane portion induces membrane fusion occurring due to spike protein cleavage through transmembrane protease serin 2 and cathepsin B and L [16-18]. This would help determine the infectivity and pathogenesis of the infection [19-21]. Similar to the high expression of the ACE2 receptor in cardiac cells, studies also found different virus particles that enabled direct activation of myocardial inflammation [22-24].

From the data above we can postulate this liability of developing incident cardiac abnormalities after a respiratory infection due to coexisting systematic inflammation [25]. As a predecessor of atheromas, it can easily progress to a thrombotic or embolic phenomenon [26]. Widespread activation of the inflammation induces maladaptive remodeling in different organs including the heart. This leads to raised levels of cytokines precipitating a "cytokine storm" that causes organ damage. Positive cases for COVID-19 infection reported elevated levels of several biomarkers such as interleukin-2,-6,-7, C-reactive protein, tumor necrosis factor α, monocyte chemoattractant protein 1, macrophage inflammatory protein 1-α, interferon-γ inducible protein-10, procalcitonin, and ferritin [27,28]. A study was found to show that patients with HF and COVID-19 had higher blood levels of cytokines IL-10 and TNF-α indicating widespread systemic inflammation [29]. Other studies have found interleukin-6 to promote its prothrombotic state and enhance its role in platelets and antibody formation, which resulted in myocardial inflammation [30]. There have been cytokines like interleukin-1 reported in the setting of acute HF and myocarditis activated by intense NOD, LRR, pyrin domain-protein 3 inflammasome formation [31]. These elevated levels of cytokines potentiate derangements in electrolytes such as calcium, potassium, and connexin 43 on cells bordering the infarct zone. This alters the normal action potential duration in cardiac myocytes, resulting in conduction abnormalities [32,33]. Following this, Rey et al. identified atrial fibrillation as the strong predictor of acute onset HF. Atrial fibrillation is associated with systemic inflammation, which can trigger detrimental alterations in the heart's structure and function. These changes include fibrosis and blood clots, where excessive fibrous tissue develops in the heart's atria, impairing their ability to contract properly, and blood clots may break loose and travel to other parts of the body, potentially causing blockages in blood vessels. Over time, these factors can contribute to the development of HF [34].

Clinical features

The virus spreads from person to person via respiratory droplets produced when infected individuals cough, sneeze, or talk. Not all infected persons are symptomatic, as some persons with positive SARS-CoV-2 tests show no symptoms of COVID-19. Once exposed, the incubation period of this disease is about four to five days. WHO reported that 80-85% of positive COVID-19 cases typically manifest as asymptomatic or mild, with most cases lasting only one to two weeks with flu-like symptoms [35-39]. When symptomatic, patients infected with SARS-CoV-2 present with symptoms including fever, sore throat, cough, loss of taste and smell, diarrhea, muscle or body aches, and loss of voice [36]. Patients with long COVID experience one or more of these symptoms for weeks to months, where symptoms persist for weeks or months after the acute phase of a COVID-19 infection has resolved. Long COVID affects different body systems, which leads to various adverse outcomes such as cardiovascular, thrombotic, neurological, and cerebrovascular diseases.

HF in COVID-19 patients presents with heterogeneous clinical symptoms, including shortness of breath, fatigue, chest pain, palpitations, dizziness, fatigue, dyspnea on exertion, cough, weight gain, abdominal bloating, leg swelling, and impaired activity tolerance. This underscores the importance of meticulous history taking in prompt diagnosis of HF. Diagnosis can be particularly challenging in patients with long COVID as there is significant overlap in signs and symptoms of HF. HF presents with clear criteria such as fluid overload and exacerbations. However, long COVID is characterized by a constellation of neurocognitive and autonomic symptoms; some long COVID symptoms, such as fatigue and shortness of breath, can mimic those of HF. Fatigue in long COVID is often debilitating and persistent, impacting daily functioning and quality of life. Shortness of breath, another common symptom, can be exertional or even present at rest, resembling the dyspnea observed in HF patients [37].

Diagnosis

Rohun et al. describe three cases that developed HF with varied presentations secondary to myocarditis from COVID-19. In these patients, there were classic findings of HF such as presentations fitting the New York Heart Association classification and elevated levels of BNP and NT proBNP, Kerley B lines on chest x-ray, and confirmatory findings on echocardiography and cardiac MRI. ECHO findings showed left ventricular ejection fraction as low as 18-48% and decreased global longitudinal strains between -13 and -7 for the left ventricle [35]. Other studies developed similar criteria for new-onset HF with major criteria as no former history of HF with either two of the three findings such as presenting symptoms of congestion, elevated levels of BNP >100 mg/dL, or NT-proBNP >300 mg/dL, and chest x-ray signs of HF or echocardiographic evidence of diastolic or systolic myocardial dysfunction [5]. The practicality of elevated NT-proBNP was assessed in de-novo HF cases and found to be significant for mortality by 36.4% with around 33% lesser ICU or ventilator-free days [40].

On echocardiography, right ventricular abnormalities were most commonly seen during the acute phase of infection, and remodeling of the right ventricle was associated with increased mortality by about two-fold [41]. These abnormalities are said to have arisen from the increased stress provided by respiratory distress [41-44]. Li et al. also suggest that a decrease in right ventricular strain is the optimal echocardiographic marker for death during hospitalization, invasive ventilation, or acute HF [42]. The ECHOVID-19 trial assessed biventricular function through echocardiography markers during the acute phase and follow-up of COVID-19 infection. While the right ventricular function seemed to improve, left ventricular dysfunction was persistent post-infection, and there was a comparatively negligible improvement in biventricular function [45,46].

There are reports of structural modifications to the myocardium seen on follow-up of patients following a COVID-19 infection. About 78% of patients who recovered from infection had cardiac abnormalities and 60% demonstrated inflammation on T1 and T2 enhancement of cardiac MRI [47]. Studies have also found reports of late gadolinium enhancement on CMR in 38% of these patients [48]. CMR can show myocardial edema, pericardial enhancement along with myocardial inflammation [49]. Viewing positive inflammatory findings or scarring on CMR in COVID-19-causing myocardial dysfunction is an indication of impending HF [50]. Positive emission tomography was also found to show cardiac abnormalities in these COVID-19 patients with negative post-acute sequelae of SARS-CoV-2 [47,51].

Management

The approach to treating HF in long COVID is like that of HF, which involves managing presenting symptoms and preventing complications from the disease. The 2021 ESC Guidelines on HF provide a Class I recommendation for the treatment of all HFrEF patients, which is the combination of any of the following drugs: angiotensin-converting enzyme (ACE) inhibitor, or angiotensin receptor-neprilysin inhibitor (ARNI), a beta-blocker, a mineralocorticoid receptor antagonist (MRA), and a sodium-glucose co-transporter 2 (SGLT2) inhibitor (dapagliflozin or\and empagliflozin). An ARNI can also be the first-line treatment instead of an ACE inhibitor. It is important to initiate treatment as soon as possible based on this recommended protocol. While new studies suggest that beta-blockers and SGLT2 inhibitors could also be used as first-line treatment, there is not yet sufficient evidence to support this approach. The RECOVERY trial labeled tocilizumab effective in improving survival outcomes in COVID-19 patients with hypoxia requiring ventilatory support and is a component of COVID-19 therapy recommended guidelines [52,53]. Coyle et al. could successfully resolve a patient's acute HF with the administration of a single dose of 4 mg/kg intravenous monoclonal antibody tocilizumab (interleukin-6 inhibitor) along with high-dose corticosteroids within 24 hours of observation [6]. They also added aldose reductase inhibitor AT-001, proven to provide mortality benefit in animal models, to attenuate inflammatory complications from fulminant myocarditis [6,54]. Similarly, Chitturi et al. resolved biventricular HF and cardiogenic shock and cytokine storm using tocilizumab and vasoactive supportive medications. They noted the higher efficacy of this drug that avoided invasive therapies such as extracorporeal membrane oxygenation and mechanical circulatory support [55]. There have been reports of interleukin-1 inhibitors (anakinra) for HF or myocarditis but they have not been reported in cases of COVID-19-associated HF [31].

For patients with overt HF, long-term management includes implantation of left ventricular assist devices or even heart transplantation. Sripanthong et al. recommend standard goal-mediated therapy in cases of severe left ventricular systolic dysfunction; however, the benefits have not been studied yet [50].

As these patients follow HF protocol for management, there is also an emphasis on delivering personalized therapy based on patient presentation and risk stratification [56]. Physicians should be vigilant in monitoring blood volume, especially for right HF, controlled fluid infusion volume, heart rate, and pulmonary venous pressure monitoring. For cases of decompensated failure, Zhou et al. defer medical treatment and focus on supportive therapy with mechanical ventilation and extracorporeal membrane oxygenation [57]. This also highlights the importance of primary prevention of SARS-CoV-2 infection and COVID-19, which can help restrain the inevitable effects of HF [12].

Banerjee also recommended attention to the structural and intermediate determinants of health equity that harness the psychosocial and environmental stressors and as a result, impact the biological and psychological well-being of patients experiencing sequelae of long COVID [58]. 

## Conclusions

The emergence of HF as a complication of long COVID has been observed over time, and despite extensive research on COVID-19, the current understanding of its pathophysiology and treatment options is still limited. This has resulted in long COVID becoming a significant public health concern. To improve the health outcomes of patients, early detection of COVID-19 and thorough cardiac evaluations are recommended. Given the impact of long COVID on various bodily systems, a multidisciplinary approach is essential for the effective treatment of HF caused by long COVID.
